# Novel apparatus for the automationof solvent extraction

**DOI:** 10.1155/S1463924681000230

**Published:** 1981

**Authors:** John G. Williams, Peter B. Stockwell, Michael Holmes, Derrick G. Porter

**Affiliations:** 1 Laboratory of the Government Chemist Stamford St. London SE1 9NQ UK; 2 Plasma Therm Ltd. 6 Station Road Penge UK


greater than the monochromator's specification, it should be noted that the monochromator used in this study is over ten years old. Because this error is a rather smooth and stable function of wavelength, it is possible to calculate the wavelength of an unknown emission line in a complex sample to an accuracy better than the monochromator specification. The measured wavelength is slightly dependent on temperature, as shown in Table 4. Thus, for the best accuracy, the monochromator should be calibrated at the temperature at which it is to be used. Also, data can be collected for a few points in the region of interest, and the wavelength shift can be calculated for the operating temperature by comparing these data to the calibration values at a standard temperature. The microprocessor controller enables the overall performance of the monochromator to be improved beyond its basic specifications, as well as providing the automation features.

*Software for the control programs, artwork for the PC boards and complete documentation are available from the authors.*

**Table 4. Wavelength accuracy reproducibility and temperature data for the EU-700 monochromator**

| Temperature (°C) | Average value | Literature value (6) | Error nm |
|---|---|---|---|
| 24.0 | 253.54 | 253.65 | −0.11 |
| | 365.06 | 365.01 | +0.05 |
| | 404.73 | 404.66 | +0.07 |
| | 435.90 | 435.84 | +0.06 |
| | 546.10 | 546.08 | +0.02 |
| 25.1 | 253.55 | 253.65 | −0.10 |
| | 365.09 | 365.01 | +0.08 |
| | 404.77 | 404.66 | +0.11 |
| | 435.91 | 435.84 | +0.07 |
| | 546.12 | 546.08 | +0.04 |
| 27.1 | 253.57 | 253.65 | −0.08 |
| | 365.10 | 365.01 | +0.09 |
| | 404.77 | 404.66 | +0.11 |
| | 435.92 | 435.84 | +0.08 |
| | 546.13 | 546.08 | +0.05 |

REFERENCES

[1] GCA Corporation, "Scanning Monochromator EU-700 and EUE-700 Series", Acton, MA, USA, 1968.

[2] Fractional and Subfractional Horsepower Electric Motors. C.G. Veinott, McGraw-Hill Book Company, New York, 1970.

[3] Cordos, E., and Malmstadt, H.V., (1975) *Anal. Chem.* **45**, 425(2), "Programmable Monochromator for Accurate High Speed Wavelength Isolation".

[4] Avery, J., Ph D. Thesis, University of Illinois, School of Chemical Sciences, Urbana, IL, 1978.

[5] Lovse, D., Ph D. Thesis, University of Illinois, School of Chemical Sciences, Urbana, IL, 1977.

[6] The Chemical Rubber Company, Handbook of Chemistry and Physics, Cleveland, OH, 1970, p E-220.

### Novel apparatus for the automation of solvent extraction

**John G. Williams, Peter B. Stockwell*, Michael Holmes, Derrick G. Porter.**
*Laboratory of the Government Chemist, Stamford St., London SE1 9NQ, UK.*

### Introduction

Chemical analyses usually require pretreatment of the sample prior to measurement. When solutions are employed as transport media for the analytes, convenient minimisation of physical and chemical interferences may be brought about by solvent extraction. Previously, automatic solvent extraction systems have been based on manual techniques, whereby the eye of the human operator is replaced by some form of phase boundary detector and the tap of the separating funnel is replaced by an electromechanical valve. Most phase boundary sensors have broadly similar characteristics. Apart from operating problems they all rely on the differential transmission of electromagnetic radiation on either side of the boundary. Trowell has described phase boundary detectors based on differential conductivity [1] and differential capacitance [2]. Associated with the latter, relying on a dielectric change, are methods using a change of refractive index [3] to control a bistable valve as an interface flows past a fixed point in the system. Recent, unpublished work performed at this Laboratory has shown that ultrasonic transducers are also capable of phase boundary detection.

Other well tried forms of solvent extraction (other than chromatographic) involve the migration of the species of interest across a semi-permeable membrane under the influence of either a concentration gradient or a potential gradient, or a combination of the two. Methods relying on the gravity separation of two completely immiscible phases are sometimes employed in continuous-flow air-segmented analytical systems; when well designed they are relatively trouble free. Vallis [4] designed a somewhat different approach to automated solvent extraction based on a rotatable cup-shaped vessel with a porous lid attached to the lip. The cup is placed inside a collecting vessel; if the porous lid is made from hydrophilic material such as sintered glass, water will pass into the collecting vessel at low rotation speeds leaving the organic phase in the cup. An increased rotation speed then ejects the organic phase. The use of a hydrophobic material such as sintered PTFE enables the preferential rejection of the organic phase.

A prototype separator which has been designed and built at this Laboratory using a completely new approach, is currently the subject of a patent application [5]. In principle, separation is effected by absorption of both phases into a porous nickel-chrome alloy disc mounted on a motor-driven shaft. Controlled angular acceleration and centripetal force on the droplets within the pores enables one phase to be separated from the other. The speed of rotation of the porous disc is coupled microelectronically to the vertical component of its motion so that separated droplets leaving the disc tangentially are trapped by hitting the walls of

*Present address: Plasma Therm Ltd., 6 Station Road, Penge, UK.

concentrically arranged glass vessels. Valves are provided at the base of each system so that separated droplets may be removed for further processing. By applying a potential between the rotating disc and a rigid electrode situated about 5 mm from the edge of the rotor, a current can be sensed as soon as the speed of rotation has increased sufficiently to effect spin-off of liquid droplets. This signal may be used to instruct the motor to continue to run at a constant speed. Alternative provisions are included to enable the conditions required for droplet throw-off to be electronically memorised. Continuous extraction is effected by the repetitive up and down motion of the spinning disc. It is at present designed for the separation of two liquid phases although provision for a third phase is easily added.

### Apparatus

The apparatus is shown in Figure 1. The body of the extraction vessel is made of Pyrex glass. Separation is effected by absorption of a batch containing both phases into a porous 2 cm diameter nickel-chrome alloy disc (A) the upper surface of which is domed. The disc is mounted on the end of a stainless steel shaft (B) turned by a geared high torque electric motor. The disc-shaft-motor assembly can be transported along its axis of rotation to any of three stations. The assembly is shown at its bottom station, with the porous disc within the inner vessel (C), around which is a collar (D) forming the first annular pocket (E). The collar itself forms the inner wall of the second annular pocket (F), the outer wall of which extends upwards to support a Perspex lid (G). The inner vessel and both annular pockets are fitted with drain valves. A stiff piece of platinum wire passes through the lid into the glassware as far as the level of the first annular pocket.

In operation, the mixed liquid to be separated is pumped into the vessel, covering the disc at its bottom station. The disc is set to spin at high speed, thoroughly mixing the liquid. The spinning of the disc is stopped and the disc raised electromechanically to a position just above the top of the upstanding collar. At the same time, the motor starts to spin the disc, the speed of which is smoothly increased until droplets of the first phase come off and a significant current flow is observed between the rotating disc and the platinum wire. The rotor continues to spin at a constant speed for fifteen seconds, sufficient time for the first phase to be thrown off the disc. The disc is then raised to its top station and accelerated, throwing off the aqueous phase. The rotating disc remains in this position for a further fifteen seconds after which it returns to the lower position. The process is then repeated. The linear electromechanical actuator and the motor used for spinning the disc were both obtained from Portescap (U.K.) Ltd. of Reading. Either phase may be selected for one hundred percent purity by adjusting the sensitivity of the droplet detector. In general, the second phase is 70%-75% pure. The apparatus has been used with several solvent combinations including chloroform/water.

The nickel chrome alloy is available from Dunlop Aviation Group and has the trade name of "Retimet". Retimet is suitable although its affinity for the organic phase is greater than for the aqueous phase; this can be used to advantage. However, in order to produce approximately equal affinity for each phase the discs were gold plated for ten minutes using a current of 300 mA. It is preferable that the alloy is shaped so that the pore structure is maintained on the surface after machining. Spark erosion is found to be successful and is available from Pantograph Precision Ltd, Slough. An alternative material for the disc is vitreous carbon. Suitable pore sizes depend on the solvents to be separated but that corresponding to 60 pores per linear inch is appropriate for most of the common laboratory solvents.

The solvent separation presumably depends on the combined effects of density and relative affinities of the porous disc for each solvent. Table 1 illustrates the relative affinity for various solvents for the case of a one phase system. The figures given represent the amount of each solvent withdrawn by a porous disc from a reservoir after being submerged each time to a constant depth. The disc used for these simple experiments was as supplied by the manufacturers prior to plating.

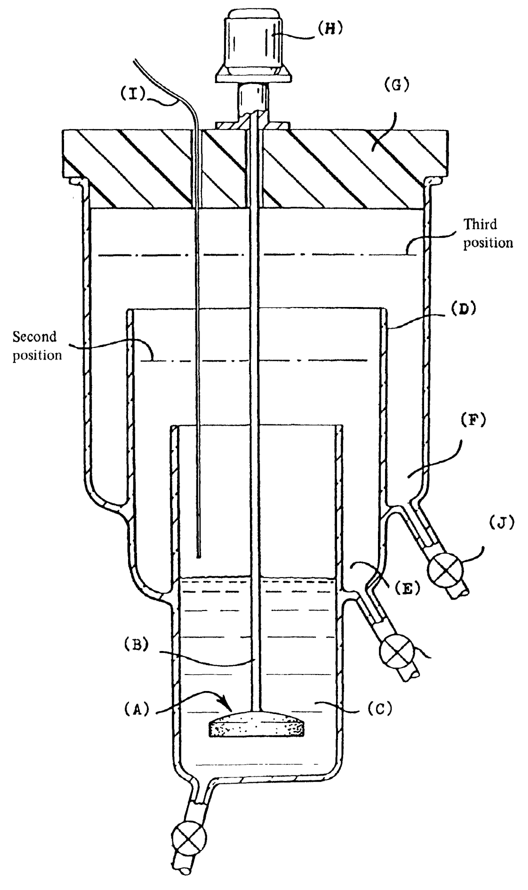

*Figure 1. Schematic layout of centrifugal separation system.*

### Electronic control

The electronic control provides accurate repeatable control of the angular acceleration of the porous disc and controls the upward and downward movement of the disc after timed intervals in the cycle. A microprocessor-based system was chosen as this provides the necessary accuracy by the employment of digital techniques whereby all the timed periods are derived from the quartz crystal controlled clock of the microprocessor. The microprocessor approach also allows versatility as any modifications can be made simply by altering the program rather than by redesigning the circuit board. This aspect was found particularly useful during the development stage.

**Table 1. Results obtained at room temperature.**
Mass of porous disc used : 3.33 g.

| Solvent | Amount withdrawn (mmoles) |
|---|---|
| Water | 66± 3 |
| n-Hexane | 20± 1 |
| trichloromethane | 24± 2 |
| 2-propanone | 35± 3 |
| diethyl ether | 25± 2 |
| glycerol | 32± 10 |
| ethanol | 46± 1 |

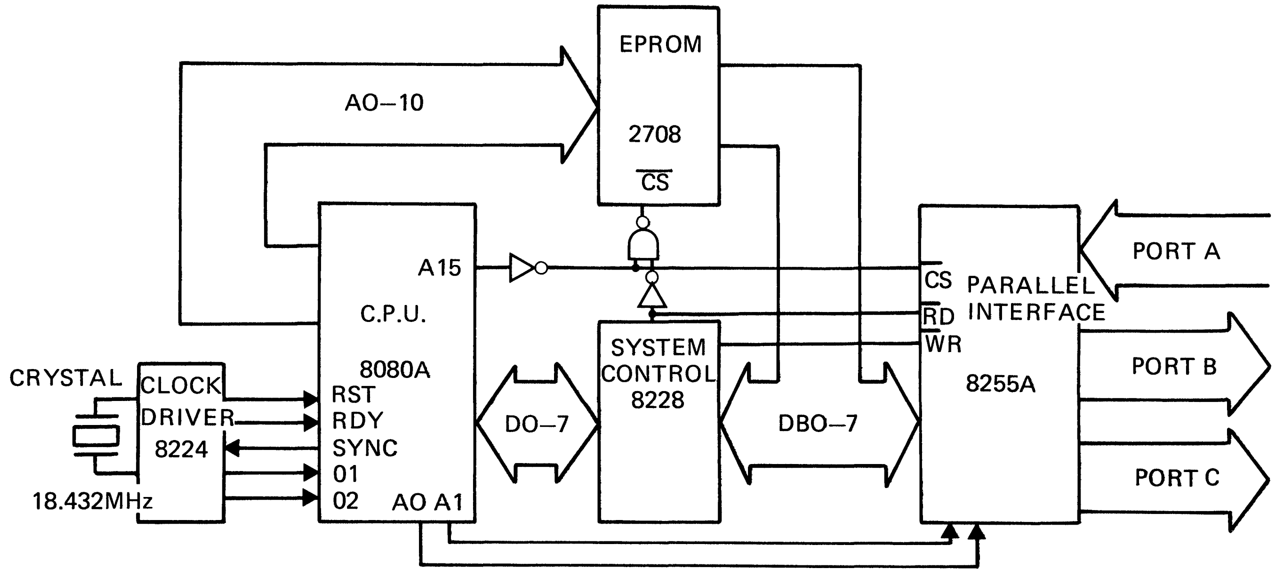

*Figure 2. Microprocessor board.*

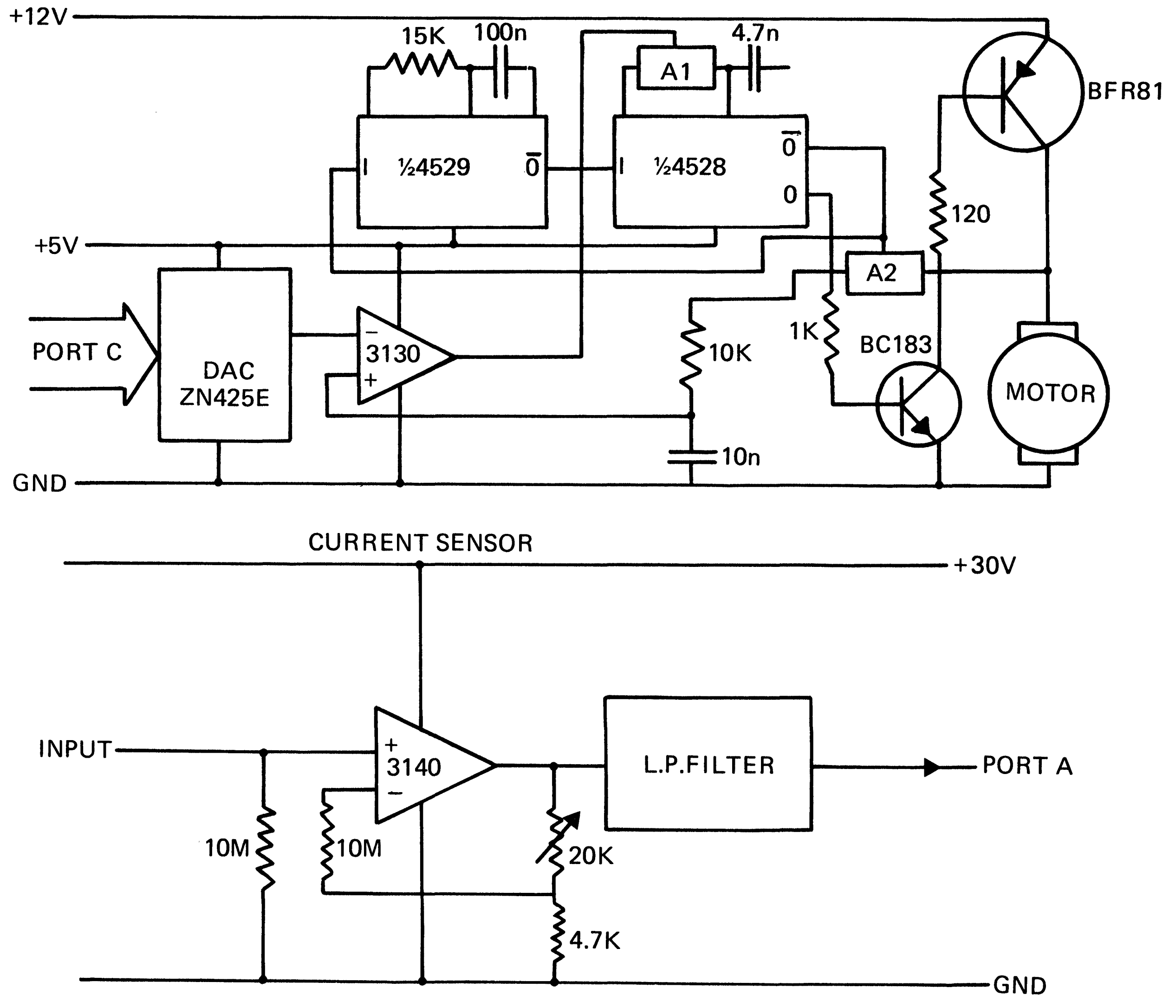

*Figure 3. Motor speed control.*

The electronic system of the apparatus can be divided into two main parts: (i) The microprocessor board consisting of the central processing unit (CPU), erasable programmable read only memory (EPROM) and the associated peripheral devices. (ii) The Control board consisting of the digital to analogue converter (DAC), motor controller, droplet detector current sensor and the relays.

The microprocessor board, shown in Figure 2, is based on the Intel 8080A CPU and its associated peripheral devices. The program is stored in the 2708 EPROM and uses approximately 300 of the 1024 bytes available. No random access memory is used as the limited amount of data handling required can be accomplished using the six internal registers of the CPU. Communication between the microprocessor board and the rest of the system is via the 8255 programmable parallel interface which is programmed to provide sixteen outputs and eight inputs. Seven of the outputs from port C of the 8255 are used to provide data inputs for the DAC on the control board. The other output is used to activate the relay which raises the actuator. Port B provides seven outputs to the displays which give an indication of the speed, the other output being used to lower the disc at the end of the cycle. Of the eight inputs to port A, one is used for the output from the circuit which senses the current produced when drops spin off, the other seven inputs are used to select a preset speed when the droplet sensor is not used. By activating these inputs one of seven preset speeds stored in the memory can be selected.

The control board is shown in Figure 3. The speed control data from the microprocessor board is converted by the DAC into a reference voltage for the motor control circuit. A Ferranti ZN425E DAC is used as this device provides a voltage output as opposed to the current output of most other devices and is substantially cheaper. The bit seven input of the DAC is not used, bit eight activates the full speed stirring which mixes the two phases together at the bottom of the apparatus. The least significant six bits are used gradually to increase the speed in sixty increments at the rate of one per second. This produces reproducibly uniform acceleration of the disc.

The motor speed control circuit operates by pulsing the motor on and off. This approach allows feedback of the motor speed to be obtained by sensing the back e.m.f. during the period when the motor is turned off. During this off period, which is fixed at 1.3ms, the motor is connected to capacitor C by the analogue switch (A2). The back e.m.f. is retained on the capacitor during the on period of the motor and compared with the voltage output from the DAC. The output of the comparator controls the analogue switch (A1). This switch operates in a quasi-linear mode and varies the length of the on period to keep the back e.m.f. (and hence the speed) close to the reference voltage supplied by the DAC. The method used in this circuit of only varying the on period to provide speed control is preferable to other methods as it allows smooth control at slow speeds. This is because the total on/off period is shortest at slow speeds i.e. when the on period is small.

The current detector circuit is necessary to detect the current of a few nanoamps which flows in the presence of drops when a potential of 30V is applied between the disc and the detector wire. The circuit uses a CA3140 MOS input operational amplifier with a typical input current of 10pA at 25 degrees centigrade. The sensitivity of this circuit can be as low as 1nA and is adjustable. To prevent false triggering by electrical interference, two more operational amplifiers are used to produce a fourth order low pass filter with a cut-off frequency of 10Hz. In addition, a small amount of software filtering has been incorporated.

The program was written in assembly language and consists mainly of timing loops with various breakpoints where operations are performed such as activating the actuator or incrementing the motor speed and displays.

REFERENCES

[1] Trowell, F. (1969) *Laboratory Practice,* **18**, 44.
[2] Trowell, F. (1967) U.K. Pat. Application, 17329/67.
[3] Porter, D.G., Jackson, C.J., Bunting, W., (1974) *Laboratory Practice,* **23**, 111.
[4] Vallis, D.G., (1967) U.K. Pat. Application, 14964/67.
[5] Williams, J.G., Stockwell, P.B. (1980) U.K. Pat. Application, 8023547.

